# *N*-(3,4-Dimethyl­phen­yl)succinamic acid

**DOI:** 10.1107/S1600536810002084

**Published:** 2010-01-23

**Authors:** B. Thimme Gowda, Sabine Foro, B. S. Saraswathi, Hartmut Fuess

**Affiliations:** a Department of Chemistry, Mangalore University, Mangalagangotri 574 199, Mangalore, India; b Institute of Materials Science, Darmstadt University of Technology, Petersenstrasse 23, D-64287 Darmstadt, Germany

## Abstract

The asymmetric unit of the title compound, C_12_H_15_NO_3_, contains two independent mol­ecules. In both mol­ecules, the conformations of the amide oxygen and the carbonyl O atom of the acid segment are *anti* to the adjacent CH_2_ groups. In the crystal, both molecules form inversion dimers linked by pairs of O—H⋯O hydrogen bonds and N—H⋯O interactions link the dimers into [100] chains.

## Related literature

For the crystal structures of related anilides, see: Gowda *et al.* (2007 ▶); Gowda, Foro, Saraswathi & Fuess (2009 ▶); Gowda, Foro, Saraswathi *et al.* (2009 ▶). For the modes of inter­linking carboxylic acids by hydrogen bonds, see: Leiserowitz (1976 ▶); Jagannathan *et al.* (1994 ▶).
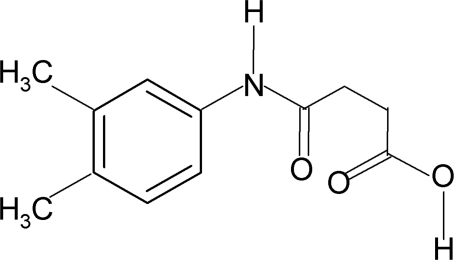


## Experimental

### 

#### Crystal data

C_12_H_15_NO_3_
*M* *_r_* = 221.25
Triclinic, 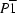

*a* = 9.736 (1) Å
*b* = 9.919 (1) Å
*c* = 12.601 (2) Å
α = 106.42 (1)°
β = 100.98 (1)°
γ = 99.81 (1)°
*V* = 1112.9 (2) Å^3^
*Z* = 4
Mo *K*α radiation
μ = 0.10 mm^−1^
*T* = 299 K
0.44 × 0.40 × 0.16 mm

#### Data collection

Oxford Diffraction Xcalibur diffractometer with a Sapphire CCD detector
Absorption correction: multi-scan (*CrysAlis RED*; Oxford Diffraction, 2009 ▶) *T*_min_ = 0.959, *T*_max_ = 0.985
7776 measured reflections
4538 independent reflections
3173 reflections with *I* > 2σ(*I*)
*R*_int_ = 0.013

#### Refinement

*R*[*F* ^2^ > 2σ(*F* ^2^)] = 0.042
*wR*(*F* ^2^) = 0.118
*S* = 1.03
4538 reflections
305 parameters
2 restraints
H atoms treated by a mixture of independent and constrained refinement
Δρ_max_ = 0.17 e Å^−3^
Δρ_min_ = −0.17 e Å^−3^

### 

Data collection: *CrysAlis CCD* (Oxford Diffraction, 2009 ▶); cell refinement: *CrysAlis RED* (Oxford Diffraction, 2009 ▶); data reduction: *CrysAlis RED*; program(s) used to solve structure: *SHELXS97* (Sheldrick, 2008 ▶); program(s) used to refine structure: *SHELXL97* (Sheldrick, 2008 ▶); molecular graphics: *PLATON* (Spek, 2009 ▶); software used to prepare material for publication: *SHELXL97*.

## Supplementary Material

Crystal structure: contains datablocks I, global. DOI: 10.1107/S1600536810002084/rz2408sup1.cif

Structure factors: contains datablocks I. DOI: 10.1107/S1600536810002084/rz2408Isup2.hkl

Additional supplementary materials:  crystallographic information; 3D view; checkCIF report

## Figures and Tables

**Table 1:** Hydrogen-bond geometry (Å, °)

*D*—H⋯*A*	*D*—H	H⋯*A*	*D*⋯*A*	*D*—H⋯*A*
O3—H3*O*⋯O5^i^	0.85 (2)	1.82 (2)	2.6681 (18)	174 (2)
N1—H1*N*⋯O4^ii^	0.894 (18)	2.127 (18)	2.9909 (18)	162.5 (15)
O6—H6*O*⋯O2^i^	0.88 (2)	1.78 (2)	2.6555 (18)	175 (2)
N2—H2*N*⋯O1	0.87 (2)	2.26 (2)	3.0676 (19)	154.7 (17)

Symmetry codes: (i) 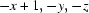; (ii) 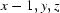.

**Fig. 1.**
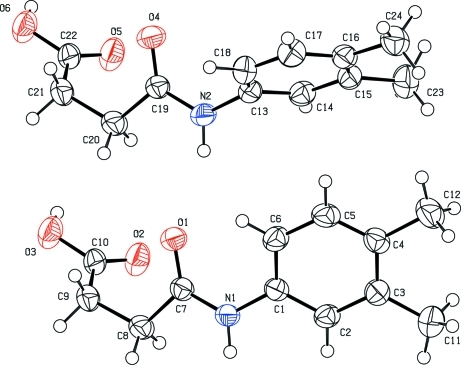
The molecular structure of the title compound, showing the atom labelling scheme. Displacement ellipsoids are drawn at the 50% probability level. The H atoms are represented as small spheres of arbitrary radii.

**Fig. 2.**
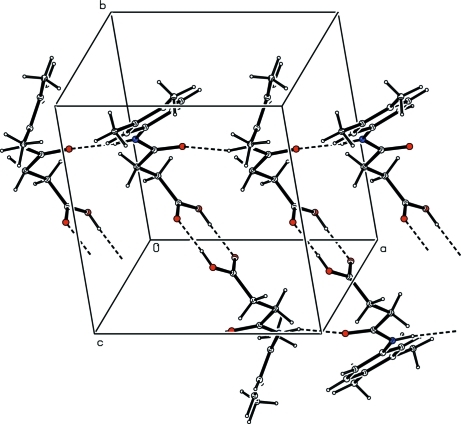
Molecular packing of the title compound with hydrogen bonding shown as dashed lines.

## supplementary crystallographic information

### Comment

As a part of studying the effect of the ring and side chain substitutions on the crystal structures of anilides (Gowda *et al.*, 2007; Gowda, Foro, Saraswathi & Fuess, 2009; Gowda, Foro, Saraswathi *et al.*, 2009), we report herein the crystal structure of *N*-(3,4-dimethylphenyl)succinamic acid (I). The asymmetric unit of (I) contains two independent molecules (Fig. 1). The conformations of the N—H and C═O bonds in the amide segments are *anti* to each other. Further, the conformation of the amide oxygen and the carbonyl oxygen of the acid segment are *anti* to the H atoms of their adjacent CH_2_ groups, while the conformation of the C=O and O—H bonds of the acid group are in *syn* position to each other, similar to that observed in *N*-(3,4-dichlorophenyl)succinamic acid monohydrate (II) (Gowda, Foro, Saraswathi & Fuess, 2009) and *N*-(2,6-dimethylphenyl)succinamic acid (III) (Gowda, Foro, Saraswathi *et al.*, 2009).

The conformation of the amide hydrogen in (I) is *syn* to the *meta*-methyl group in the benzene ring, contrary to the *anti* conformation observed between the amide hydrogen and the *meta*-Cl in (II). Further, the conformation of the amide oxygen and the carbonyl oxygen of the acid segment are *syn* to each other, contrary to the *anti* conformation observed in (II). N—H···O and O—H···O intermolecular hydrogen bonds pack the molecules into chains running parallel to the *a* axis (Table 1, Fig. 2).

The modes of interlinking carboxylic acids by hydrogen bonds is described elsewhere (Leiserowitz, 1976). The packing of molecules involving dimeric hydrogen bonded association of each carboxyl group with a centrosymmetrically related neighbor has also been observed (Jagannathan *et al.*, 1994).

### Experimental

A solution of succinic anhydride (0.02 mol) in toluene (25 ml) was treated dropwise with a solution of 3,4-dimethylaniline (0.02 mol) in toluene (20 ml) with constant stirring. The resulting mixture was stirred for about one hour and set aside for an additional hour at room temperature for the completion of reaction. The mixture was then treated with dilute hydrochloric acid to remove the unreacted 3,4-dimethylaniline. The resultant solid *N*-(3,4-dimethylphenyl)succinamic acid was filtered under suction and washed thoroughly with water to remove the unreacted succinic anhydride and succinic acid. It was recrystallized to constant melting point from ethanol.

The purity of the compound was checked by elemental analysis and characterized by its infrared and NMR spectra. The rod like colourless single crystals used in X-ray diffraction studies were grown by slow evaporation at room temperature of an ethanolic solution.

### Refinement

The H atoms of the NH groups were located in a difference Fourier map and their positions refined with N—H = 0.87 (2)–0.89 (2) %A. The H atoms of the OH groups were located in a difference Fourier map and the O—H distance restrained to 0.82 (2) Å. All other H atoms were positioned with idealized geometry using a riding model with C—H = 0.93–0.97 Å and refined with isotropic displacement parameters set to 1.2 times of the *U*_eq_ of the parent atom.

### Crystal data

**Table:**

C_12_H_15_NO_3_	*Z* = 4
*M**_r_* = 221.25	*F*(000) = 472
Triclinic, *P*1	*D*_x_ = 1.320 Mg m^−^^3^
Hall symbol: -P 1	Mo *K*α radiation, λ = 0.71073 Å
*a* = 9.736 (1) Å	Cell parameters from 2369 reflections
*b* = 9.919 (1) Å	θ = 3.1–27.7°
*c* = 12.601 (2) Å	µ = 0.10 mm^−^^1^
α = 106.42 (1)°	*T* = 299 K
β = 100.98 (1)°	Rod, colourless
γ = 99.81 (1)°	0.44 × 0.40 × 0.16 mm
*V* = 1112.9 (2) Å^3^	

### Data collection

**Table:**

Oxford Diffraction Xcalibur diffractometer with a Sapphire CCD detector	4538 independent reflections
Radiation source: fine-focus sealed tube	3173 reflections with *I* > 2σ(*I*)
graphite	*R*_int_ = 0.013
Rotation method data acquisition using ω and φ scans.	θ_max_ = 26.4°, θ_min_ = 3.1°
Absorption correction: multi-scan (*CrysAlis RED*; Oxford Diffraction, 2009)	*h* = −11→12
*T*_min_ = 0.959, *T*_max_ = 0.985	*k* = −12→10
7776 measured reflections	*l* = −14→15

### Refinement

**Table:**

Refinement on *F*^2^	Primary atom site location: structure-invariant direct methods
Least-squares matrix: full	Secondary atom site location: difference Fourier map
*R*[*F*^2^ > 2σ(*F*^2^)] = 0.042	Hydrogen site location: inferred from neighbouring sites
*wR*(*F*^2^) = 0.118	H atoms treated by a mixture of independent and constrained refinement
*S* = 1.02	*w* = 1/[σ^2^(*F*_o_^2^) + (0.0517*P*)^2^ + 0.2735*P*] where *P* = (*F*_o_^2^ + 2*F*_c_^2^)/3
4538 reflections	(Δ/σ)_max_ < 0.001
305 parameters	Δρ_max_ = 0.17 e Å^−^^3^
2 restraints	Δρ_min_ = −0.17 e Å^−^^3^

### Special details

**Table:**

Experimental. CrysAlis RED (Oxford Diffraction, 2009) Empirical absorption correction using spherical harmonics, implemented in SCALE3 ABSPACK scaling algorithm.
Geometry. All e.s.d.'s (except the e.s.d. in the dihedral angle between two l.s. planes) are estimated using the full covariance matrix. The cell e.s.d.'s are taken into account individually in the estimation of e.s.d.'s in distances, angles and torsion angles; correlations between e.s.d.'s in cell parameters are only used when they are defined by crystal symmetry. An approximate (isotropic) treatment of cell e.s.d.'s is used for estimating e.s.d.'s involving l.s. planes.
Refinement. Refinement of *F*^2^ against ALL reflections. The weighted *R*-factor *wR* and goodness of fit *S* are based on *F*^2^, conventional *R*-factors *R* are based on *F*, with *F* set to zero for negative *F*^2^. The threshold expression of *F*^2^ > σ(*F*^2^) is used only for calculating *R*-factors(gt) *etc*. and is not relevant to the choice of reflections for refinement. *R*-factors based on *F*^2^ are statistically about twice as large as those based on *F*, and *R*- factors based on ALL data will be even larger.

### Fractional atomic coordinates and isotropic or equivalent isotropic displacement parameters (Å^2^)

**Table:**

	*x*	*y*	*z*	*U*_iso_*/*U*_eq_	
O1	0.25568 (12)	0.46649 (13)	0.13575 (10)	0.0454 (3)	
O2	0.15120 (14)	0.11617 (14)	0.05553 (10)	0.0529 (3)	
O3	0.20038 (15)	0.09014 (14)	−0.11316 (10)	0.0530 (3)	
H3O	0.243 (2)	0.030 (2)	−0.0934 (17)	0.064*	
N1	0.08150 (14)	0.53340 (15)	0.22197 (11)	0.0380 (3)	
H1N	−0.013 (2)	0.5115 (19)	0.2162 (14)	0.046*	
C1	0.16800 (16)	0.63065 (16)	0.32941 (13)	0.0344 (4)	
C2	0.11108 (17)	0.64457 (17)	0.42386 (14)	0.0382 (4)	
H2	0.0170	0.5949	0.4138	0.046*	
C3	0.19110 (18)	0.73073 (17)	0.53296 (14)	0.0395 (4)	
C4	0.33168 (18)	0.80814 (18)	0.54764 (14)	0.0409 (4)	
C5	0.38529 (18)	0.79638 (18)	0.45219 (15)	0.0436 (4)	
H5	0.4780	0.8489	0.4613	0.052*	
C6	0.30594 (17)	0.70921 (17)	0.34342 (14)	0.0409 (4)	
H6	0.3448	0.7036	0.2808	0.049*	
C7	0.12894 (16)	0.45212 (17)	0.13761 (13)	0.0344 (3)	
C8	0.01037 (17)	0.33813 (18)	0.04349 (13)	0.0404 (4)	
H8A	−0.0624	0.3841	0.0153	0.048*	
H8B	−0.0348	0.2689	0.0750	0.048*	
C9	0.06420 (18)	0.25797 (19)	−0.05577 (13)	0.0423 (4)	
H9A	−0.0173	0.2094	−0.1214	0.051*	
H9B	0.1280	0.3278	−0.0758	0.051*	
C10	0.14225 (17)	0.14906 (17)	−0.03140 (13)	0.0386 (4)	
C11	0.1276 (2)	0.7378 (2)	0.63389 (15)	0.0568 (5)	
H11A	0.0286	0.6851	0.6075	0.068*	
H11B	0.1334	0.8370	0.6751	0.068*	
H11C	0.1804	0.6960	0.6834	0.068*	
C12	0.4230 (2)	0.9026 (2)	0.66431 (15)	0.0592 (5)	
H12A	0.5170	0.9429	0.6584	0.071*	
H12B	0.4315	0.8457	0.7144	0.071*	
H12C	0.3788	0.9794	0.6945	0.071*	
O4	0.75949 (12)	0.46145 (14)	0.15126 (10)	0.0545 (4)	
O5	0.66192 (15)	0.10463 (14)	0.06567 (10)	0.0548 (3)	
O6	0.71268 (15)	0.07822 (14)	−0.10294 (10)	0.0524 (3)	
H6O	0.756 (2)	0.015 (2)	−0.0837 (17)	0.063*	
N2	0.57898 (15)	0.50597 (17)	0.23359 (12)	0.0478 (4)	
H2N	0.486 (2)	0.484 (2)	0.2237 (16)	0.057*	
C13	0.66100 (17)	0.61991 (18)	0.33598 (13)	0.0390 (4)	
C14	0.66191 (17)	0.60174 (18)	0.44074 (14)	0.0398 (4)	
H14	0.6180	0.5122	0.4434	0.048*	
C15	0.72698 (17)	0.71425 (18)	0.54223 (13)	0.0385 (4)	
C16	0.79482 (17)	0.84812 (17)	0.53795 (14)	0.0387 (4)	
C17	0.79743 (19)	0.86189 (18)	0.43201 (15)	0.0448 (4)	
H17	0.8453	0.9495	0.4286	0.054*	
C18	0.73127 (19)	0.7499 (2)	0.33125 (14)	0.0457 (4)	
H18	0.7342	0.7623	0.2612	0.055*	
C19	0.63180 (17)	0.43683 (17)	0.14927 (13)	0.0363 (4)	
C20	0.51791 (18)	0.32399 (19)	0.05010 (14)	0.0434 (4)	
H20A	0.4690	0.2529	0.0782	0.052*	
H20B	0.4470	0.3706	0.0201	0.052*	
C21	0.57788 (19)	0.24675 (19)	−0.04634 (13)	0.0441 (4)	
H21A	0.4993	0.1993	−0.1143	0.053*	
H21B	0.6441	0.3182	−0.0629	0.053*	
C22	0.65414 (17)	0.13717 (17)	−0.02138 (13)	0.0389 (4)	
C23	0.7220 (2)	0.6912 (2)	0.65422 (15)	0.0559 (5)	
H23A	0.6822	0.5908	0.6411	0.067*	
H23B	0.6628	0.7483	0.6900	0.067*	
H23C	0.8177	0.7199	0.7031	0.067*	
C24	0.8606 (2)	0.9755 (2)	0.64499 (15)	0.0536 (5)	
H24A	0.9336	0.9518	0.6945	0.064*	
H24B	0.7873	0.9991	0.6833	0.064*	
H24C	0.9029	1.0570	0.6255	0.064*	

### Atomic displacement parameters (Å^2^)

**Table:**

	*U*^11^	*U*^22^	*U*^33^	*U*^12^	*U*^13^	*U*^23^
O1	0.0328 (6)	0.0520 (7)	0.0422 (7)	0.0089 (5)	0.0099 (5)	0.0014 (5)
O2	0.0701 (9)	0.0541 (8)	0.0409 (7)	0.0255 (7)	0.0226 (6)	0.0134 (6)
O3	0.0680 (9)	0.0579 (8)	0.0407 (7)	0.0301 (7)	0.0231 (6)	0.0127 (6)
N1	0.0285 (7)	0.0405 (8)	0.0367 (7)	0.0091 (6)	0.0057 (6)	0.0011 (6)
C1	0.0318 (8)	0.0303 (8)	0.0355 (8)	0.0101 (7)	0.0051 (6)	0.0031 (7)
C2	0.0330 (8)	0.0350 (8)	0.0421 (9)	0.0073 (7)	0.0106 (7)	0.0054 (7)
C3	0.0425 (9)	0.0358 (9)	0.0386 (9)	0.0148 (7)	0.0092 (7)	0.0073 (7)
C4	0.0394 (9)	0.0362 (9)	0.0393 (9)	0.0115 (7)	0.0026 (7)	0.0039 (7)
C5	0.0324 (8)	0.0384 (9)	0.0493 (10)	0.0036 (7)	0.0069 (7)	0.0032 (8)
C6	0.0382 (9)	0.0389 (9)	0.0419 (9)	0.0079 (7)	0.0134 (7)	0.0062 (7)
C7	0.0327 (8)	0.0348 (8)	0.0335 (8)	0.0098 (7)	0.0056 (6)	0.0085 (7)
C8	0.0340 (8)	0.0410 (9)	0.0378 (9)	0.0092 (7)	0.0041 (7)	0.0032 (7)
C9	0.0414 (9)	0.0450 (10)	0.0316 (8)	0.0101 (8)	0.0041 (7)	0.0022 (7)
C10	0.0394 (9)	0.0354 (9)	0.0303 (8)	0.0037 (7)	0.0069 (7)	−0.0012 (7)
C11	0.0591 (12)	0.0626 (13)	0.0442 (11)	0.0140 (10)	0.0158 (9)	0.0091 (9)
C12	0.0544 (12)	0.0601 (12)	0.0453 (11)	0.0113 (10)	−0.0011 (9)	0.0001 (9)
O4	0.0315 (6)	0.0638 (8)	0.0517 (8)	0.0084 (6)	0.0116 (5)	−0.0053 (6)
O5	0.0720 (9)	0.0607 (8)	0.0413 (7)	0.0297 (7)	0.0245 (6)	0.0165 (6)
O6	0.0651 (9)	0.0557 (8)	0.0399 (7)	0.0249 (7)	0.0220 (6)	0.0093 (6)
N2	0.0291 (7)	0.0580 (9)	0.0406 (8)	0.0045 (7)	0.0091 (6)	−0.0046 (7)
C13	0.0322 (8)	0.0439 (9)	0.0348 (9)	0.0094 (7)	0.0093 (7)	0.0029 (7)
C14	0.0379 (9)	0.0368 (9)	0.0439 (9)	0.0088 (7)	0.0129 (7)	0.0105 (7)
C15	0.0391 (9)	0.0415 (9)	0.0354 (8)	0.0146 (7)	0.0099 (7)	0.0099 (7)
C16	0.0355 (8)	0.0386 (9)	0.0384 (9)	0.0113 (7)	0.0075 (7)	0.0065 (7)
C17	0.0470 (10)	0.0371 (9)	0.0489 (10)	0.0056 (8)	0.0138 (8)	0.0136 (8)
C18	0.0479 (10)	0.0553 (11)	0.0353 (9)	0.0117 (9)	0.0126 (8)	0.0159 (8)
C19	0.0325 (8)	0.0383 (9)	0.0351 (8)	0.0104 (7)	0.0072 (7)	0.0071 (7)
C20	0.0353 (9)	0.0443 (10)	0.0405 (9)	0.0095 (7)	0.0049 (7)	0.0016 (8)
C21	0.0449 (9)	0.0467 (10)	0.0313 (9)	0.0100 (8)	0.0037 (7)	0.0029 (7)
C22	0.0380 (9)	0.0377 (9)	0.0309 (8)	0.0038 (7)	0.0072 (7)	−0.0005 (7)
C23	0.0693 (13)	0.0605 (12)	0.0423 (10)	0.0198 (10)	0.0161 (9)	0.0195 (9)
C24	0.0522 (11)	0.0458 (10)	0.0491 (11)	0.0100 (9)	0.0061 (9)	−0.0003 (9)

### Geometric parameters (Å, °)

**Table:**

O1—C7	1.2233 (18)	O4—C19	1.2194 (18)
O2—C10	1.2203 (19)	O5—C22	1.221 (2)
O3—C10	1.3106 (19)	O6—C22	1.3115 (19)
O3—H3O	0.850 (15)	O6—H6O	0.875 (15)
N1—C7	1.3500 (19)	N2—C19	1.334 (2)
N1—C1	1.4224 (19)	N2—C13	1.434 (2)
N1—H1N	0.894 (18)	N2—H2N	0.87 (2)
C1—C6	1.385 (2)	C13—C18	1.376 (2)
C1—C2	1.388 (2)	C13—C14	1.381 (2)
C2—C3	1.389 (2)	C14—C15	1.388 (2)
C2—H2	0.9300	C14—H14	0.9300
C3—C4	1.401 (2)	C15—C16	1.399 (2)
C3—C11	1.506 (2)	C15—C23	1.500 (2)
C4—C5	1.383 (2)	C16—C17	1.384 (2)
C4—C12	1.506 (2)	C16—C24	1.503 (2)
C5—C6	1.386 (2)	C17—C18	1.381 (2)
C5—H5	0.9300	C17—H17	0.9300
C6—H6	0.9300	C18—H18	0.9300
C7—C8	1.514 (2)	C19—C20	1.517 (2)
C8—C9	1.517 (2)	C20—C21	1.517 (2)
C8—H8A	0.9700	C20—H20A	0.9700
C8—H8B	0.9700	C20—H20B	0.9700
C9—C10	1.489 (2)	C21—C22	1.487 (2)
C9—H9A	0.9700	C21—H21A	0.9700
C9—H9B	0.9700	C21—H21B	0.9700
C11—H11A	0.9600	C23—H23A	0.9600
C11—H11B	0.9600	C23—H23B	0.9600
C11—H11C	0.9600	C23—H23C	0.9600
C12—H12A	0.9600	C24—H24A	0.9600
C12—H12B	0.9600	C24—H24B	0.9600
C12—H12C	0.9600	C24—H24C	0.9600
			
C10—O3—H3O	108.4 (14)	C22—O6—H6O	109.0 (14)
C7—N1—C1	126.35 (13)	C19—N2—C13	125.70 (14)
C7—N1—H1N	116.8 (11)	C19—N2—H2N	117.3 (13)
C1—N1—H1N	115.6 (11)	C13—N2—H2N	116.9 (13)
C6—C1—C2	119.26 (14)	C18—C13—C14	119.74 (15)
C6—C1—N1	122.85 (14)	C18—C13—N2	120.78 (15)
C2—C1—N1	117.89 (14)	C14—C13—N2	119.36 (15)
C1—C2—C3	121.68 (15)	C13—C14—C15	121.39 (16)
C1—C2—H2	119.2	C13—C14—H14	119.3
C3—C2—H2	119.2	C15—C14—H14	119.3
C2—C3—C4	119.12 (15)	C14—C15—C16	119.12 (15)
C2—C3—C11	120.02 (16)	C14—C15—C23	119.71 (16)
C4—C3—C11	120.84 (15)	C16—C15—C23	121.16 (15)
C5—C4—C3	118.47 (15)	C17—C16—C15	118.37 (15)
C5—C4—C12	120.40 (16)	C17—C16—C24	120.35 (16)
C3—C4—C12	121.13 (16)	C15—C16—C24	121.26 (15)
C4—C5—C6	122.41 (16)	C18—C17—C16	122.22 (16)
C4—C5—H5	118.8	C18—C17—H17	118.9
C6—C5—H5	118.8	C16—C17—H17	118.9
C1—C6—C5	119.02 (15)	C13—C18—C17	119.08 (16)
C1—C6—H6	120.5	C13—C18—H18	120.5
C5—C6—H6	120.5	C17—C18—H18	120.5
O1—C7—N1	124.17 (14)	O4—C19—N2	123.43 (15)
O1—C7—C8	121.87 (14)	O4—C19—C20	122.88 (14)
N1—C7—C8	113.96 (13)	N2—C19—C20	113.68 (14)
C7—C8—C9	113.04 (13)	C21—C20—C19	113.63 (14)
C7—C8—H8A	109.0	C21—C20—H20A	108.8
C9—C8—H8A	109.0	C19—C20—H20A	108.8
C7—C8—H8B	109.0	C21—C20—H20B	108.8
C9—C8—H8B	109.0	C19—C20—H20B	108.8
H8A—C8—H8B	107.8	H20A—C20—H20B	107.7
C10—C9—C8	113.76 (14)	C22—C21—C20	114.09 (14)
C10—C9—H9A	108.8	C22—C21—H21A	108.7
C8—C9—H9A	108.8	C20—C21—H21A	108.7
C10—C9—H9B	108.8	C22—C21—H21B	108.7
C8—C9—H9B	108.8	C20—C21—H21B	108.7
H9A—C9—H9B	107.7	H21A—C21—H21B	107.6
O2—C10—O3	122.68 (16)	O5—C22—O6	123.06 (16)
O2—C10—C9	123.86 (15)	O5—C22—C21	123.93 (15)
O3—C10—C9	113.46 (15)	O6—C22—C21	113.01 (15)
C3—C11—H11A	109.5	C15—C23—H23A	109.5
C3—C11—H11B	109.5	C15—C23—H23B	109.5
H11A—C11—H11B	109.5	H23A—C23—H23B	109.5
C3—C11—H11C	109.5	C15—C23—H23C	109.5
H11A—C11—H11C	109.5	H23A—C23—H23C	109.5
H11B—C11—H11C	109.5	H23B—C23—H23C	109.5
C4—C12—H12A	109.5	C16—C24—H24A	109.5
C4—C12—H12B	109.5	C16—C24—H24B	109.5
H12A—C12—H12B	109.5	H24A—C24—H24B	109.5
C4—C12—H12C	109.5	C16—C24—H24C	109.5
H12A—C12—H12C	109.5	H24A—C24—H24C	109.5
H12B—C12—H12C	109.5	H24B—C24—H24C	109.5
			
C7—N1—C1—C6	33.1 (2)	C19—N2—C13—C18	63.8 (2)
C7—N1—C1—C2	−145.84 (16)	C19—N2—C13—C14	−120.24 (19)
C6—C1—C2—C3	−2.7 (2)	C18—C13—C14—C15	2.9 (2)
N1—C1—C2—C3	176.29 (14)	N2—C13—C14—C15	−173.11 (15)
C1—C2—C3—C4	1.5 (2)	C13—C14—C15—C16	−1.0 (2)
C1—C2—C3—C11	−177.34 (15)	C13—C14—C15—C23	178.29 (15)
C2—C3—C4—C5	0.4 (2)	C14—C15—C16—C17	−1.6 (2)
C11—C3—C4—C5	179.24 (16)	C23—C15—C16—C17	179.16 (16)
C2—C3—C4—C12	−179.75 (16)	C14—C15—C16—C24	177.00 (15)
C11—C3—C4—C12	−0.9 (3)	C23—C15—C16—C24	−2.2 (2)
C3—C4—C5—C6	−1.1 (3)	C15—C16—C17—C18	2.3 (3)
C12—C4—C5—C6	179.05 (16)	C24—C16—C17—C18	−176.32 (16)
C2—C1—C6—C5	2.0 (2)	C14—C13—C18—C17	−2.2 (2)
N1—C1—C6—C5	−176.97 (15)	N2—C13—C18—C17	173.74 (15)
C4—C5—C6—C1	−0.1 (3)	C16—C17—C18—C13	−0.4 (3)
C1—N1—C7—O1	−10.9 (3)	C13—N2—C19—O4	1.7 (3)
C1—N1—C7—C8	168.62 (15)	C13—N2—C19—C20	−178.33 (16)
O1—C7—C8—C9	−6.0 (2)	O4—C19—C20—C21	−0.8 (2)
N1—C7—C8—C9	174.42 (14)	N2—C19—C20—C21	179.30 (15)
C7—C8—C9—C10	75.78 (18)	C19—C20—C21—C22	75.46 (19)
C8—C9—C10—O2	6.6 (2)	C20—C21—C22—O5	4.0 (2)
C8—C9—C10—O3	−173.82 (14)	C20—C21—C22—O6	−176.04 (14)

### Hydrogen-bond geometry (Å, °)

**Table:**

*D*—H···*A*	*D*—H	H···*A*	*D*···*A*	*D*—H···*A*
O3—H3O···O5^i^	0.85 (2)	1.82 (2)	2.6681 (18)	174 (2)
N1—H1N···O4^ii^	0.894 (18)	2.127 (18)	2.9909 (18)	162.5 (15)
O6—H6O···O2^i^	0.88 (2)	1.78 (2)	2.6555 (18)	175 (2)
N2—H2N···O1	0.87 (2)	2.26 (2)	3.0676 (19)	154.7 (17)

Symmetry codes: (i) −*x*+1, −*y*, −*z*; (ii) *x*−1, *y*, *z*.
